# High adsorption to methylene blue based on Fe_3_O_4_–N-banana-peel biomass charcoal†

**DOI:** 10.1039/d4ra04973j

**Published:** 2024-08-15

**Authors:** Zhu-Xiang Gong, Mfitumucunguzi Steven, Yan-Ting Chen, Li-Zhu Huo, Hao Xu, Chao-Fei Guo, Xue-Juan Yang, Yu-Xuan Wang, Xi-Ping Luo

**Affiliations:** a College of Chemistry and Materials Engineering, Zhejiang A&F University Hangzhou 311300 China 20190050@zafu.edu.cn luoxiping@zafu.edu.cn; b Zhejiang Provincial Key Laboratory of Chemical Utilization of Forestry Biomass Hangzhou 311300 China

## Abstract

This research focused on utilizing banana peel as the primary material for producing mesoporous biomass charcoal through one-step potassium hydroxide activation. Subsequently, the biomass charcoal underwent high-temperature calcination with varying impregnation ratios of KOH : BC for different durations in tubular furnaces set at different temperatures. The resultant biomass charcoal was then subjected to hydrothermal treatment with FeCl_3_·6H_2_O to produce biochar/iron oxide composites. The adsorption capabilities of these composites towards methylene blue (MB) were examined under various conditions, including pH (ranging from 3 to 12), temperature variations, and initial MB concentrations (ranging from 50 to 400 mg L^−1^). The adsorption behavior aligned with the Langmuir model and demonstrated quasi-secondary kinetics. After five adsorption cycles, the capacity decreased from 618.64 mg g^−1^ to 497.18 mg g^−1^, indicating considerable stability. Notably, Fe_3_O_4_–N-BC exhibited exceptional MB adsorption performance.

## Abstract 2

This research focused on utilizing banana peel as the primary material for producing mesoporous biomass charcoal through one-step potassium hydroxide activation.

## Graphical abstract

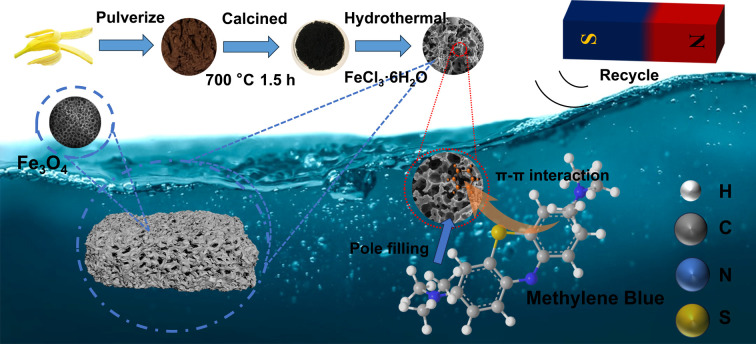

## Introduction

According to statistics, more than 10 000 commercial dye products are introduced into the aquatic environment. The textile industry is a primary contributor to water pollution, alongside significant sectors such as paint, leather, paper, and printing industries.^1,2^ Methylene blue (MB) stands as a representative cationic organic dye, commonly utilized in various industries such as papermaking, silk, cotton, wool, and hair coloring. With the economy experiencing rapid growth, industrial production processes are yielding increasingly large volumes of dye effluent, predominantly composed of organic compounds like azo, amino, and phenyl compounds. The discharge of wastewater containing dyes not only pollutes the aquatic environment but also disrupts the ecological balance of aquatic life and poses risks to human health.^3^ Elevated levels of MB in water can lead to noticeable changes in color and turbidity, along with potential skin irritation and neurological effects. Prolonged consumption of water contaminated with dyes poses a severe risk to human health, causing significant damage to the liver and digestive system over time.^4^ Therefore, it is imperative to implement appropriate measures to remove MB from dye wastewater, ensuring both water quality preservation and the safeguarding of human health from contamination. Reports indicate the frequent utilization of various methods for remediating dyes in contaminated wastewater, including Fenton oxidation,^5^ photocatalytic oxidation,^6^ membrane separation,^7^ adsorption,^8^ and electrochemical approaches.^9^ Compared to other approaches, the adsorption method has advantages. It is one of the most extensively used wastewater treatment technologies due to its simple design, high regeneration potential, diverse material source, good performance, and cost-effectiveness.^10^

Activated carbon (AC) is a widely utilized adsorbent in wastewater treatment technology owing to its expansive specific surface area, well-suited pore distribution, and robust mechanical and thermal stability.^11^ However, most AC on the market today is made from coal, lignite, and coking coal as precursors. These resources have the disadvantage of being costly and non-renewable.^12^ Compared to coal, a non-renewable resource for carbon production, biomass carbon sources are both renewable and widely available. The utilization of biomass energy can reduce dependence on fossil fuels, thereby mitigating air and water pollution. Due to its larger pore structure and more active sites, biomass carbon exhibits enhanced adsorption capacity, which has garnered significant attention from researchers. Research has demonstrated that BC effectively eliminates hazardous cationic dyes from wastewater.^13^ Crop straws, forestry and agricultural wastes, coal, and municipal sewage can all be used to make BC.^14^ It can accomplish the recycling and reuse of waste resources in addition to lowering the pollution that rubbish burning and decomposition contribute to the environment. To obtain greater adsorption effects, biomass charcoal's chemical and physical characteristics must be enhanced. The original biomass charcoal's adsorption capacity to remove contaminants from wastewater is still restricted. The primary objective of modification in biomass charcoal is to enhance its specific surface area and pore structure, while concurrently enriching the material's surface functional groups. Additionally, it is crucial for biomass charcoal to facilitate easy separation into solid and liquid components. The recent surge in popularity of iron modification of biomass charcoal is attributed to its ability to introduce magnetic components, thereby facilitating efficient solid–liquid separation.^15^ Furthermore, adding iron to biomass charcoal can increase its surface area and create a pore structure, resulting in more active sites for adsorption.^16^ Nevertheless, the application of iron oxide onto biomass charcoal may potentially occupy adsorption sites or impede the pore structure, consequently diminishing its adsorption capacity.^17^ Nitrogen doping is a promising modifying technique. Nitrogen dopants can increase the electrical conductivity of the carbon matrix, resulting in more active catalytic sites.^18–20^ Nitrogen-modified biomass charcoal has been frequently employed in catalysis because of its local unpaired electrons.^21^ It has also been discovered that nitrogen alteration can diminish the surface area and pore volume of biomass charcoal.^22^ Consequently, the advantages of enhanced surface area, functional groups, and magnetic components are obtained by iron-nitrogen co-doping. The adsorption mechanisms of iron-nitrogen co-modified biomass charcoal for the removal of pollutants may involve π–π stacking, surface complexation, hydrogen bonding, and pore diffusion.^23^ Numerous studies have explored Fe–N modified biomass charcoal as an adsorbent. For instance, Zhou *et al.* pioneered the development of urea-functionalized magnetic biomass charcoal, employing it as an adsorbent for the extraction of Pb.^24^ Ai *et al.* produced NH_4_Cl-induced magnetic buckwheat husk powder biomass charcoal and utilized it to adsorb tetracycline and Zn(ii).^25^ According to the literature, practically all adsorbent modification materials based on biomass charcoal are carbonized before being activated with an activator. This approach is not only energy-intensive, but it is also complex and time-consuming, and the adsorbed biomass charcoal is difficult to recover.^26^ Some adsorbents have low adsorption capacity due to their small specific surface area; some adsorbents have problems such as difficulty in separation and recovery; magnetic modification is an excellent solution to their separation and recovery; however, magnetic modification generally leads to a decrease in the adsorbent's adsorption capacity, so novel adsorption materials must be developed to maintain a magnetic adsorbent while also having a high adsorption capability. FeCl_3_ can be used as a magnetic source to improve the separation and recovery capacity of biochar due to its low cost and low pollution.

In this study, biomass charcoal was prepared with banana peels as precursors and potassium hydroxide as an activator. To investigate the impact of temperature, activator ratio, and carbonization period on adsorption performance, an orthogonal experiment was created. To make nitrogen-modified biomass charcoal (N-BC), find the ideal preparation conditions, combine it with urea in these settings, and pyrolyze. To manufacture magnetic Fe_3_O_4_, utilize a hot solvent approach. Then, dope Fe_3_O_4_ with biomass charcoal to create Fe_3_O_4_–N-BC, which is very valuable, recyclable, and makes the adsorbent magnetic to lower secondary recycling costs. The iron-nitrogen co-modification method presents several advantages, including the augmentation of surface area, enhancement of functional groups, and introduction of magnetic components, potentially rendering it a feasible alternative for biochar preparation. To explore the impacts of Fe and N modification on the morphology, chemical composition, and functional groups of biomass charcoal, the sample underwent characterization using scanning electron microscopy (SEM), X-ray diffraction (XRD), X-ray photoelectron spectroscopy (XPS), and vibrating-sample magnetometry (VSM).

## Materials and methods

### Materials and reagents

Banana peels were purchased from Zhejiang Province, China. Ethylene glycol, potassium hydroxide (KOH), and absolute ethanol were all of analytical grade and purchased from Macklin (Shanghai, China). Hydrochloric acid (HCl, 36–38 wt%) and sodium hydroxide (NaOH) were all of analytical grade and purchased from Sinopharm Group Chemical Reagent Co., Ltd (Shanghai, China), FeCl_3_·6H_2_O (99%), sodium acetate trihydrate (NaAc·3H_2_O) (99%), urea (99%), methylene blue (C_16_H_18_N_3_SCl) (98%) are purchased from Macklin (Shanghai, China), deionized water (18.25 MΩ cm) was prepared in the laboratory.

### Preparation of adsorbent

#### Preparation of modified biomass charcoal

The banana peels underwent a series of preparatory steps: they were initially washed with deionized water, dried, and then ground through a 40-mesh sieve using a grinder. Subsequently, 2.5 g of banana peel powder was mixed with varying proportions of KOH, followed by the addition of a specific quantity of urea. The mixture was then combined with deionized water, dried in an oven at 105 °C for 24 hours, and subsequently transferred into a tube furnace. The sample was subjected to a heating rate of 5 °C min^−1^ under a nitrogen atmosphere of 400 mL min^−1^, reaching a final temperature of 700 °C with an isothermal hold of 90 minutes. Following cooling, residual KOH was removed by washing the samples with HCl, followed by several rinses with deionized water until a neutral pH was attained. The resulting product was then dried in an oven at 80 °C for 24 hours to yield nitrogen-modified biomass charcoal (N-BC).

Because of the intricate interplay among reaction conditions, pinpointing suitable synthesis parameters proves challenging through single reaction conditions alone. Hence, an orthogonal design method was employed to elucidate the impact of each individual response factor.^27,28^ The orthogonal design table is shown in the Table S1 (ESI),† which mainly discusses the effects of biomass precursor type, carbonization time, carbonization temperature, and activator dosage on synthesis.

#### Preparation of Fe_3_O_4_-modified biomass charcoal

**Fig. 1:**
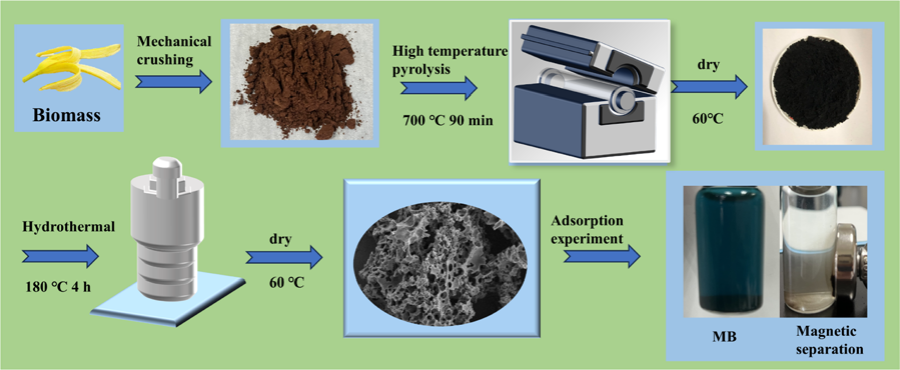
The schematic representation of the synthesis of Fe_3_O_4_–N-BC.

A total of 16.32 g of NaAc·3H_2_O and 6.54 g of FeCl_3_·6H_2_O were added to 70 mL of ethylene glycol solution and subjected to magnetic stirring until completely dissolved. The resulting solution was then poured into a 100 mL reaction kettle and heated to 180 °C for 12 h. After the reaction is completed, it is washed with deionized water and absolute ethanol in sequence, and then vacuum dried at 60 °C to obtain Fe_3_O_4_. Then, 0.5 g of the prepared Fe_3_O_4_ and 0.3 g of the biomass charcoal prepared in the optimal group of the orthogonal experiment were uniformly dispersed into 70 mL of deionized water using ultrasound, and then the Fe_3_O_4_ and N-BC mixture was poured into a 100 mL reactor and reacted at 180 °C for 4 hours. The synthesized material was subjected to magnetic separation after cooling, followed by rinsing with anhydrous ethanol and deionized water, and subsequent vacuum drying at 60 °C to yield the iron oxide and nitrogen co-doped biochar (Fe_3_O_4_–N-BC). The synthesis diagram of Fe_3_O_4_–N-BC is shown in Fig. 1.

#### Adsorption experiment

Firstly, methylene blue standard solution (1000 mg L^−1^) was prepared with deionized water, and all methylene blue solutions were prepared according to this standard solution. HCl and NaOH of 0.1 mol L^−1^ were prepared to adjust pH. In this experiment, the concentration of dye solution was determined by ultraviolet spectrophotometer at the maximum absorption wavelength *λ* = 664 nm (Fig. S1, ESI†). In this research, three adsorption tests were conducted on each experimental sample and the average value was taken.

##### Adsorption kinetics

5 mg adsorbent was mixed in methylene blue solution of 25 mL 300 mg L^−1^, and adsorption experiments were carried out at 25 °C and pH natural conditions at different times. Then the dynamic model is used to fit the data.

##### Adsorption isotherm

Dispersed 5 mg of adsorbent in 25 mL MB solutions of different concentrations, adsorb under natural pH conditions of 25 °C, 35 °C, and 45 °C, conduct isotherm research, and then used the isotherm model to fit the relevant data.

##### Adsorption thermodynamics

A methylene blue solution, initially concentrated at 300 mg L^−1^, was chosen. Successive 25 mL dye solutions were introduced to 5 mg adsorbents under ambient pH conditions. The adsorbents underwent adsorption at temperatures of 25, 35, and 45 °C. Upon reaching adsorption equilibrium, the remaining dye solution concentration was measured, followed by thermodynamic fitting.

##### The impact of pH on adsorption performance

0.1 M HCl and 0.1 M NaOH were employed to modulate the pH of the dye solution within the pH range of 3–12 at 25 °C. Subsequently, 10 mg of adsorbent was dispersed in 25 mL of a 300 mg L^−1^ MB solution at various pH levels. Post-adsorption equilibrium, the concentration of the MB solution was ascertained.

Incorporate a specified quantity of Fe_3_O_4_–N-BC adsorbent into 25 mL of dye solutions with varying concentrations. Following adsorption at a consistent temperature for a duration, the supernatant is magnetically isolated and subjected to measurement using a UV spectrophotometer at the wavelength of maximum absorption, *λ* = 664 nm. According to the standard curve generated for the methylene blue dye (Fig. S2, ESI†). Measure the absorbance of the dye. The adsorption capacity *q*_e_ (mg g^−1^) (eqn (1)) and removal rate *R* (%) (eqn (2)) under different conditions are calculated by the following formulas:1
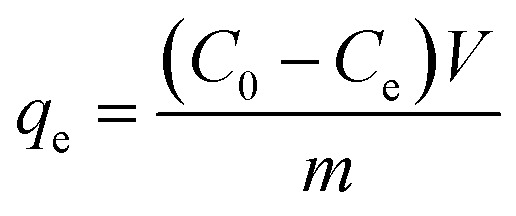
2
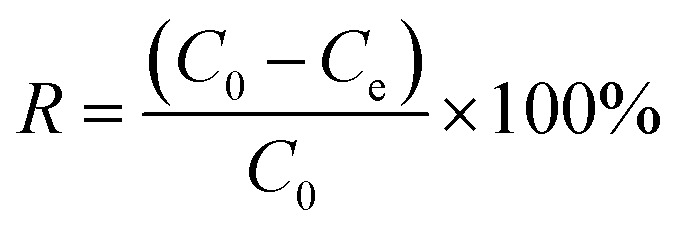
where *C*_0_ (mg L^−1^) and *C*_e_ (mg L^−1^) are the initial concentration of the dye solution and the concentration after the dye adsorption reaches equilibrium, *V* (mL) is the initial volume of the dye solution, and *m* (g) is the amount of adsorbent.

### Characterization

The sample's morphology was analyzed using scanning electron microscopy (SEM) coupled with an energy dispersive spectrometer (EDS). After the biomass charcoal was dissolved in concentrated hydrochloric acid, the content of biomass charcoal-coated iron was determined by inductively coupled plasma mass spectrometry (ICP-MS). The absorbance of residual dye in water is measured with a UV-visible spectrophotometer (UV). An X-ray diffractometer was employed to capture the inorganic crystal morphology of the sample within the range of 10° to 70°. Raman spectra were acquired utilizing a high-resolution Raman spectrophotometer from HoribaJobin-Yvon. X-ray photoelectron spectroscopy (XPS) spectra were acquired utilizing an ESCALAB250 instrument. The magnetic properties of the samples were assessed at room temperature employing a vibrating sample magnetometer (VSM, LakeShore7404). BET specific surface area measurements were performed on a Micromeritics ASAP2020 instrument (ASAP2020, Micromeritis).

## Results and discussion

### Modified synthesis results and characterizations

#### Results of orthogonal experiment

**Fig. 2:**
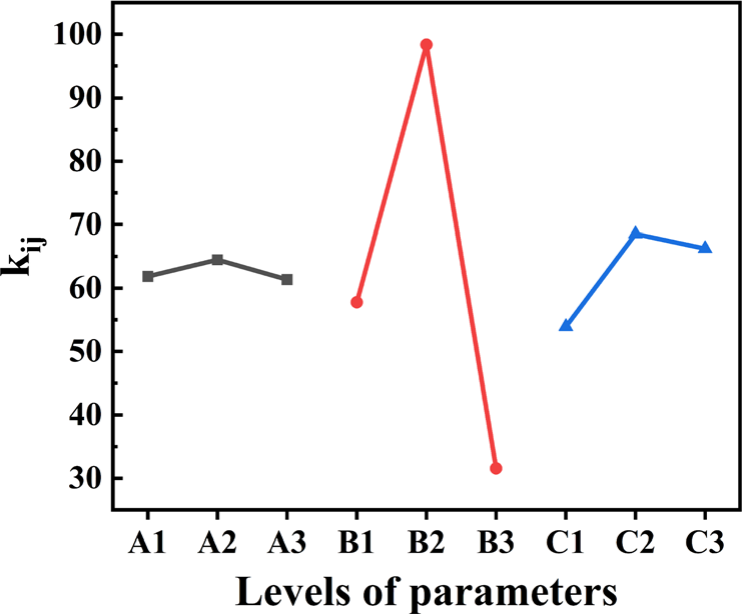
The effect of synthesis conditions on adsorption capacities: holding temperature (A), carbonization temperature (B), activator ratio (C).

The results of orthogonal experiments are depicted in Fig. 2. Here, ‘*i*’ represents the factors (*i* = A, B, C), while ‘*j*’ indicates the levels (*j* = 1, 2, 3). In detail, *k*_*ij*_ signifies the cumulative removal rate at level *j* for factor *i*, with the mean value of *k*_*ij*_ represented as *k*_*ij*_. *R*_*i*_ denotes the range between the maximum and minimum *k*_*ij*_ values across the three levels examined. The orthogonal experiments are shown in Table S2.† It can be seen from the figure that the four factors affecting the experimental conditions, carbonization temperature, holding time, biomass type and activator ratio, have an impact on the preparation of biomass charcoal: carbonization temperature > activator ratio > holding time. It can be seen that the carbonization temperature is the most important determining factor. Of course, the proportion of activators is also important. The proportion of activators added ultimately also depends on the carbonization temperature. It will affect the structure of the prepared biomass charcoal, and appropriate holding time can prevent the waste of energy and resources. The optimal levels for each factor are A_2_, B_2_, and C_2_, indicating a carbonization temperature of 700 °C, a holding time of 90 minutes, and a mass ratio of biomass to activator KOH at 1 : 2. For KOH activation,^29^ first KOH interacts with carbon components and is converted into K_2_CO_3_ (eqn (3)), then K_2_CO_3_ is decomposed into K_2_O and CO_2_ (eqn (4)), and the intermediate product further reacts with carbon to form CO (eqn (5)–(7)), the gases (H_2_O, CO_2_, CO, *etc.*) produced by the reaction help to form a porous structure. The biomass chars utilized in subsequent characterization and adsorption experiments were synthesized at their optimal levels.

3 6KOH + 2C → 2K + 3K_2_CO_3_ + 3H_2_

4 K_2_CO_3_ → K_2_O + CO_2_

5 CO_2_ + C → 2CO

6 K_2_CO_3_ + 2C → 2K + 3CO

7 K_2_O + C → 2K + CO

#### Characterization

**Fig. 3:**
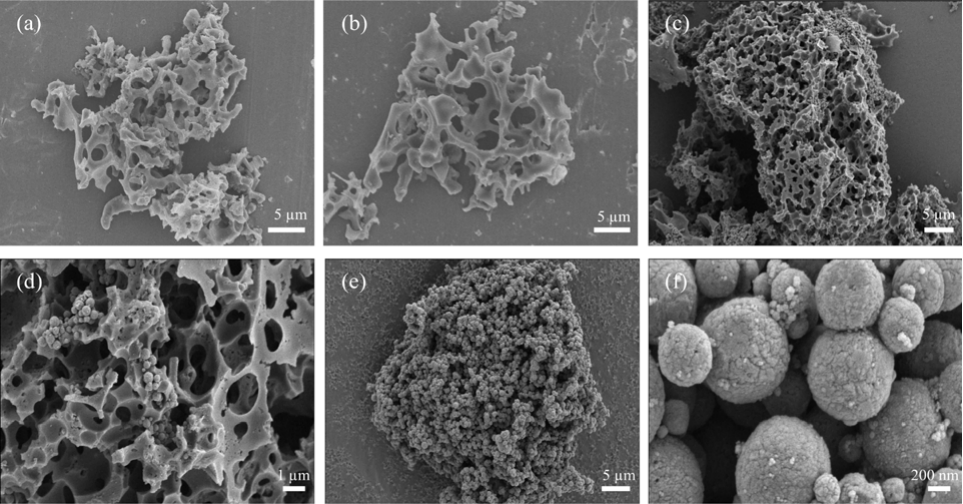
SEM images of (a) BC, (b) N-BC, (c)–(d) Fe_3_O_4_–N-BC, (e)–(f) Fe_3_O_4_.

**Fig. 4:**
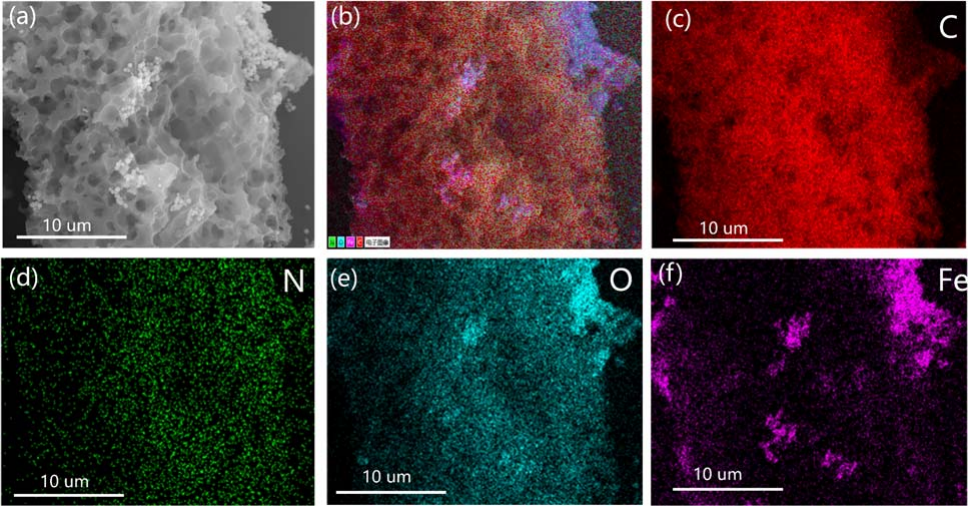
EDS diagram of Fe_3_O_4_–N-BC, (a) SEM images of Fe_3_O_4_–N-BC, (b) total element distribution, (c) C element distribution, (d) N element distribution, (e) O element distribution, (f) Fe element distribution.

The morphological characteristics of the biomass charcoal material were studied by obtaining SEM images. Fig. 3 shows the SEM images of different adsorbents BC, N-BC, and Fe_3_O_4_–N-BC. Observations from Fig. 3 (a–f) indicate that the synthesized Fe_3_O_4_ particles exhibit a spherical shape with a rough surface. Clearly depicted in the figure is the spherical Fe_3_O_4_ loaded onto the surface of biomass carbon. In order to understand the elemental composition of Fe_3_O_4_–N-BC material and the distribution of Fe_3_O_4_ on BC, the EDS-mapping spectrum was used to analyse the Fe_3_O_4_–N-BC material. Fig. 4(b)–(f) are the element mappings of C, N, O, and Fe, respectively. The distribution of the four elements can be clearly shown in the figure. At the same time, the main distribution positions of O and Fe are roughly similar, which indicates that the synthesized Fe_3_O_4_ was successfully loaded on the biomass charcoal.

**Fig. 5:**
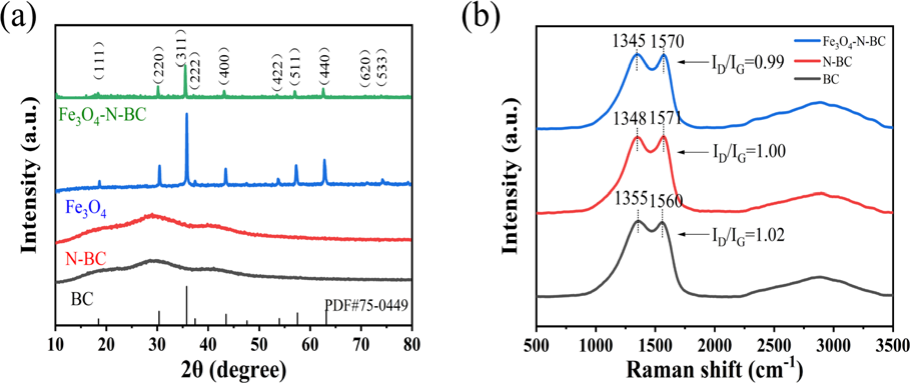
(a) XRD patterns of samples, (b) Raman spectra of N-BC and Fe_3_O_4_–N-BC.

Fig. 5(a) shows the XRD patterns of BC, N-BC, Fe_3_O_4_, and Fe_3_O_4_–N-BC. According to the standard PDF card (JCPDF #75-0449), the values of 2*θ* in the figure are at 18.4° (111), 30.3° (220), 35.7° (311), 37.4° (222), 43.4° (400), 53.9° (422), 57.5° (511), 63.1° (440), 71.6° (620), and 74.7° (533) are characteristic peaks of Fe_3_O_4_, which proves the presence of Fe_3_O_4_. Since amorphous carbon is prepared, its crystallinity is low, and broad peaks with low signal intensity appear near 28° and 40°. Because the diffraction peak intensity is weaker than that of Fe_3_O_4_, it is not obvious in the XRD pattern of Fe_3_O_4_–N-BC.^23^ The position of the characteristic peak of Fe_3_O_4_–N-BC is the same as that of Fe_3_O_4_, but the intensity of the peak is weakened. The XRD test results are related to the content of the sample. Compared with the pure Fe_3_O_4_ sample, the relative Fe_3_O_4_ content of Fe_3_O_4_–N-BC in the same amount of sample testing is reduced, so the XRD diffraction peak is relatively weakened.

In Fig. 5(b), the Raman spectrum of BC, N-BC, and Fe_3_O_4_–N-BC is presented. The D peak, observed around 1355 cm^−1^, corresponds to the disorder or defects of carbon atoms, while the G peak, positioned near 1570 cm^−1^, is associated with the surface stretching motion of sp^2^ carbon atom pairs.^30^ The two spectral bands of Fe_3_O_4_–N-BC illustrate the successful coating of carbon. The relative intensity ratio of peak D and peak G (*I*_D_/*I*_G_) indicates the degree of graphitization of the material.

The intensity ratios (*I*_D_/*I*_G_) of the D and G bands of BC, N-BC and Fe_3_O_4_–N-BC are 1.02, 1.00 and 0.99, respectively. The *I*_D_/*I*_G_ ratio serves as an indicator of the degree of graphitization within the carbon structure. A higher *I*_D_/*I*_G_ ratio suggests more pronounced defects within the carbon structure. The observed decrease in the *I*_D_/*I*_G_ value of nitrogen-modified biomass charcoal signifies the emergence of more graphitic carbon. Nitrogen modification could destroy the defects of the charcoal structure in biochar due to the reaction between the N atom and the carbon structure.^31^ A broader half-width of the band indicates an abundance of structure within disordered carbon. Following nitrogen and iron modification, notable shifts in the positions of the D and G peaks within the biochar spectrum were observed, particularly in the G peak. Consequently, nitrogen and iron modifications exert a significant influence on the structure of graphitized charcoal. Alterations in the carbon structure of biochar will inevitably impact its adsorption efficacy.

**Fig. 6:**
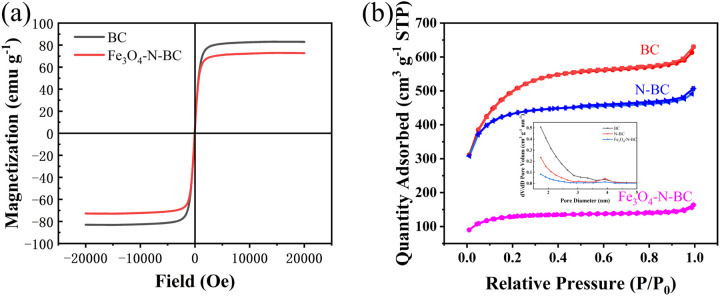
(a) Magnetic hysteresis loops of Fe_3_O_4_, Fe_3_O_4_–N-BC, (b) nitrogen adsorption–desorption isotherms and pore size distribution of BC, N-BC and Fe_3_O_4_–N-BC.

The hysteresis loops of Fe_3_O_4_ and Fe_3_O_4_–N-BC are shown in Fig. 6(a). The saturation magnetic induction intensities of Fe_3_O_4_ and Fe_3_O_4_–N-BC decrease in sequence, and their values are 82.91 emu g^−1^ and 72.68 emu g^−1^, respectively. The decrease in saturation magnetic induction intensity is due to the carbon coating of Fe_3_O_4_. In the magnetic test, the proportion of Fe_3_O_4_ decreases and its corresponding magnetic intensity decreases. In addition, ferromagnetism is characterized by remanence and saturation magnetic susceptibility (Mr/Ms). For the composite Fe_3_O_4_–N-BC (Mr/Ms = 0.06), a Mr/Ms ratio lower than 25% indicates that the synthesized Fe_3_O_4_–N-BC materials are superparamagnetic at room temperature.^32^ The magnetic property facilitates the efficient separation of the dye-adsorbed composite material from water. Hence, Fe_3_O_4_–N-BC can serve as a magnetic adsorbent for the removal of pollutants from aqueous solutions.

The porous structure of magnetic biomass charcoal composites was characterized using N_2_ adsorption/desorption isotherms. The adsorption isotherm of the biochar exhibited a typical type-I profile, characterized by a sharp increase in adsorption at *P*/*P*_0_ < 0.2, followed by saturation at relatively low relative pressures. This suggests that the biochar possesses a combined microporous and mesoporous structure.^33–35^ As shown in Fig. 6(b), according to the pore size distribution, its pore structure is mainly distributed in the range of 2–4 nm, confirming the mesoporous structure of biomass charcoal. The specific surface areas of the prepared BC, N-BC, and Fe_3_O_4_–N-BC are shown in Table S3.† Among them, BC has the largest specific surface area of 1827.9321 m^2^ g^−1^, 3.8 times that of Fe_3_O_4_–N-BC. This indicates that KOH activation significantly enhances the specific surface area of the carbon material. In contrast to BC, nitrogen modification resulted in a decrease in the surface area and total volume of biomass char, while increasing its average pore size. Nitrogen modification appears to hinder the enhancement of biochar surface area and pore structure, a phenomenon influenced by the choice of modifying reagent (urea) and pyrolysis temperature. Additionally, some studies have suggested that nitrogen modification could potentially reduce the surface area and pore volume of biochar. Consequently, employing the iron-nitrogen co-modification technique could be deemed a favorable option for biochar production owing to its capability to introduce a substantial surface area. The decrease in biochar's specific surface area following nitrogen modification may be ascribed to (1) the narrowing of pores amidst carbon fibers within the biochar, (2) the reduction/annealing process leading to the low desorption degree of biochar,^36^ (3) the modification results in highly disordered and folded structures.^22^ The introduction of Fe_3_O_4_ leads to a smaller mass proportion of biomass charcoal in the composite material, resulting in a smaller specific surface area. Iron enters the mesoporous carbon, thereby reducing the shrinkage of the matrix carbon during carbonization.^37^ But compared to other magnetic biomass charcoal, its specific surface area is still larger. KOH, urea and FeCl_3_·6H_2_O react with banana peels, and the gases generated by thermal decomposition during the reaction can improve their structural characteristics and adsorption properties.

**Fig. 7:**
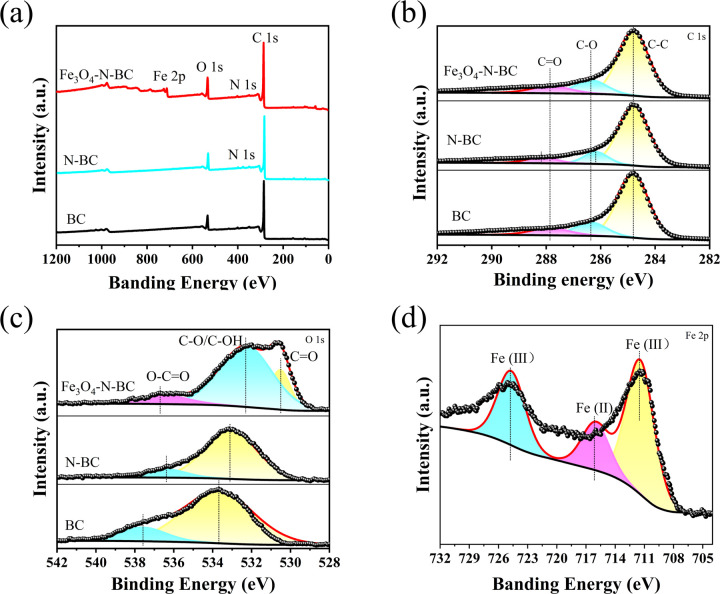
(a) XPS survey spectra and (b) high-resolution XPS spectra of C 1s (c) high-resolution XPS spectra of O 1s of BC, N-BC and Fe_3_O_4_–N-BC, (d) high-resolution XPS spectra of Fe 2p of Fe_3_O_4_–N-BC.

The XPS spectrum of biomass charcoal is shown in Fig. 7. C 1s and O 1s can be detected on the surface of biomass charcoal, while Fe 2p can also be detected on Fe_3_O_4_–N-BC. The high-resolution C 1s XPS (Fig. 7(b)) spectra in BC, N-BC and Fe_3_O_4_–N-BC can be convolved into three sub-peaks at 284.8/284.8/284.8, 286.3/286.1/286.3 and 287.8/287.1/288.1 eV, reflecting respectively C–C/C–H, C–O and O=C.^38,39^ On the O 1s spectrum (Fig. 7(c)), O=C–O (533.6/533.1 eV) exists in BC/N-BC, while in Fe_3_O_4_–N-BC, C=O (530.2 eV), C–O (531.1 eV) and O=C–O (533.4 eV) is convolved into three sub-peaks.^40,41^ After Fe modification, the peak positions of Fe 2p are 711.2, 715.7 and 724.3 eV respectively (Fig. 7(d)). 711.2 and 724.3 eV are the characteristic peaks of Fe 2p_3/2_ and Fe 2p_1/2_, respectively, and the peak at 715.7 eV is basically consistent with the Fe(iii) state.^39,42^ This shows that there are two oxidation states of iron. Combined with XRD analysis, the Fe^2+^ and Fe^3+^ compounds in biomass charcoal are magnetite (Fe_3_O_4_) and maghemite (γ-Fe_2_O_3_).^43^

#### Effects of different adsorbents on the adsorption of methylene blue

**Fig. 8:**
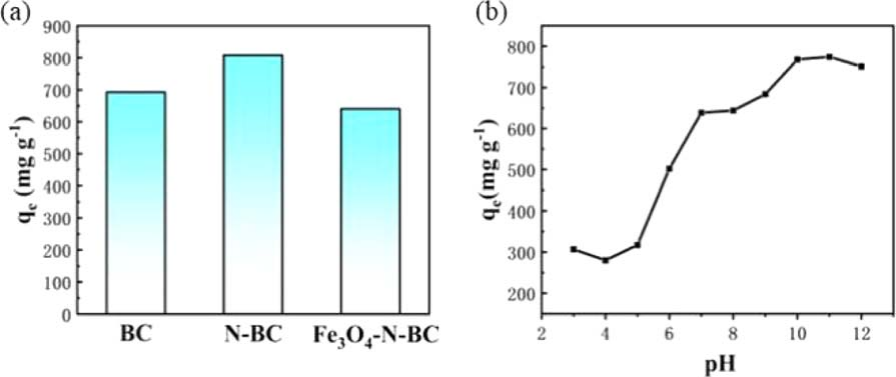
(a) Adsorption capacity of MB by BC, N-BC and Fe_3_O_4_–N-BC, (b) effect of pH on MB adsorption.

Conduct adsorption tests on methylene blue with the prepared BC, N-BC and Fe_3_O_4_–N-BC, respectively. Take 5 mg of BC, N-BC and Fe_3_O_4_–N-BC respectively and dissolve them in 25 mL of MB solution with an initial concentration of 300 mg L^−1^. The pH naturally, the temperature is 298 K. The adsorption capacity is shown in Fig. 8(a). The adsorption capacity of biomass charcoal activated by potassium hydroxide can reach 693.11 mg g^−1^, and the adsorption capacity of nitrogen-modified biomass charcoal can reach up to 807.87 mg g^−1^. The latter will cause some pores to collapse. Although the load of Fe_3_O_4_ will cause the specific surface area to decrease, the adsorption capacity is as high as 640.77 mg g^−1^. The loading of Fe_3_O_4_ results in a slight reduction in adsorption capacity, but gives it magnetism and can be recycled more conveniently. Therefore, it is valuable to sacrifice a small amount of adsorption capacity in exchange for its rapid separation capability. Later experiments were conducted on Fe_3_O_4_–N-BC.

#### Effects of pH

The pH level of the dye solution stands as a pivotal factor, wielding significant influence over the adsorption efficacy of the adsorbent. Beyond merely shaping the surface structure and charge of the biomass charcoal, its ionization level within the solution profoundly impacts the cohesion between the adsorbent and the dye molecules, thus influencing their stability. With this in mind, an investigation into the impact of pH on the removal of MB by biomass charcoal was undertaken. In the pH range of 3.0–12.0, Fe_3_O_4_–N-BC can effectively remove MB. As shown in Fig. 8(b), when the pH rises from 3 to 12, the adsorption behavior of magnetic biomass charcoal to MB first increases rapidly, then slows down, and finally remains basically unchanged. The adsorption capacity of MB at pH 3 is 306.7 mg g^−1^, to 750.8 mg g^−1^ at pH 12. The reason may be that the abundant H^+^ in the solution competes with the cationic MB for adsorption on the same active site on the adsorbent surface at an initial low pH.^44^ As the pH increases, the electrostatic adsorption of MB by the adsorbent increases, thereby improving its adsorption effect.

#### Adsorption kinetics

**Fig. 9:**
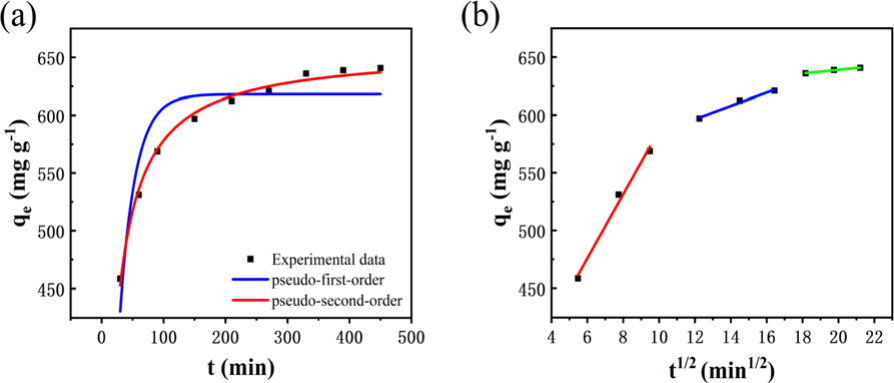
(a) Pseudo-first-order and pseudo-second-order kinetics, fitting curves of MB on Fe_3_O_4_–N-BC, (b) intra-particle diffusion model.

Adsorbents ensconced within porous materials often exhibit intricate kinetics, wherein the adsorption pace can be intricately influenced by a multitude of factors. These include the nature of the adsorbent itself, the surface uniformity or heterogeneity thereof, alongside the pH and temperature of the solution. Furthermore, the presence of characteristic functional groups plays a pivotal role in dictating the adsorption performance. In order to further determine the adsorption performance of N-BC and Fe_3_O_4_–N-BC materials on MB molecules under natural conditions, adsorption equilibrium and kinetic studies were conducted under the conditions of a natural pH value of deionized water solution and temperature of 298 K. The effect of contact time on the adsorption of MB by Fe_3_O_4_–N-BC is shown in Fig. 9(a). It can be seen that the adsorption amount increases rapidly 100 minutes before adsorption, and the adsorption capacity reached 531.17 mg g^−1^ at 60 min, and slowly increased after 200 min to finally reach adsorption equilibrium. The surface of Fe_3_O_4_–N-BC has abundant active sites and a high concentration gradient, so the adsorption speed is fast in the initial stage. When the active site surface of Fe_3_O_4_–N-BC is gradually occupied by MB, the repulsive interfacial force between the solid and liquid gradually increases, and the adsorption rate gradually decreases. In order to study the adsorption kinetics and adsorption mechanism process, three typical kinetic models^45^ (pseudo-first-order kinetics (eqn (8))), pseudo-second-order kinetics (eqn (9)) and intra-particle diffusion model (eqn (10))8
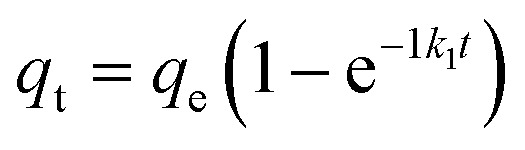
9
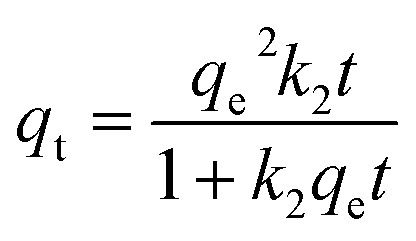
10
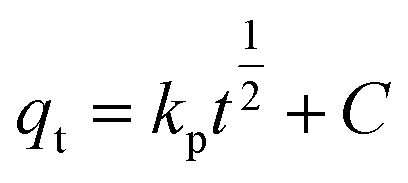
where *q*_e_ (mg g^−1^) is the equilibrium adsorption capacity, *q*_t_ (mg g^−1^) is the MB adsorption capacity at time *t*, *k*_1_ (min^−1^), *k*_2_ (g mg^−1^ min^−1^) and *k*_p_ (mg g^−1^ min^−1/2^) are the adsorption rate constant of pseudo-first-order kinetics, the adsorption constant of pseudo-second-order kinetics and the intra-particle diffusion rate constant.

Fig. 9(a) illustrates the application of nonlinear regression analysis to the dataset employing dynamic equations. The adsorption dataset underwent fitting utilizing pseudo-first-order kinetics, pseudo-second-order kinetics, and intra-particle diffusion models. Detailed parameters are delineated in Table S4.† Notably, Table S4† reveals that the *R*^2^ values for the pseudo-first-order and pseudo-second-order kinetics pertaining to methylene blue adsorption by Fe_3_O_4_–N-BC stand at 0.8316 and 0.9929, respectively. Moreover, the maximum adsorption capacities are observed to be 618.15 mg g^−1^ and 656.08 mg g^−1^ correspondingly. The adsorption of methylene blue by Fe_3_O_4_–N-BC is more consistent with the pseudo-second-order kinetic model, which indicates that the adsorption of MB on this adsorbent is chemical adsorption.^46^ As can be seen from Fig. 9(b), there are three straight lines fitting the intra-particle diffusion to the adsorption of MB on Fe_3_O_4_–N-BC. The initial stage entails the diffusion of MB from the solution to the outer surface of Fe_3_O_4_–N-BC. Subsequently, the second stage encompasses particle diffusion, wherein MB permeates from the outer surface of Fe_3_O_4_–N-BC into the inner pores. Finally, the third stage denotes the equilibrium phase. It is noteworthy that none of these three linear progressions intersects the origin, denoting C ≠ 0, thereby suggesting that particle diffusion alone does not exclusively govern the rate-limiting step.^47^

#### Adsorption isotherms

**Fig. 10:**
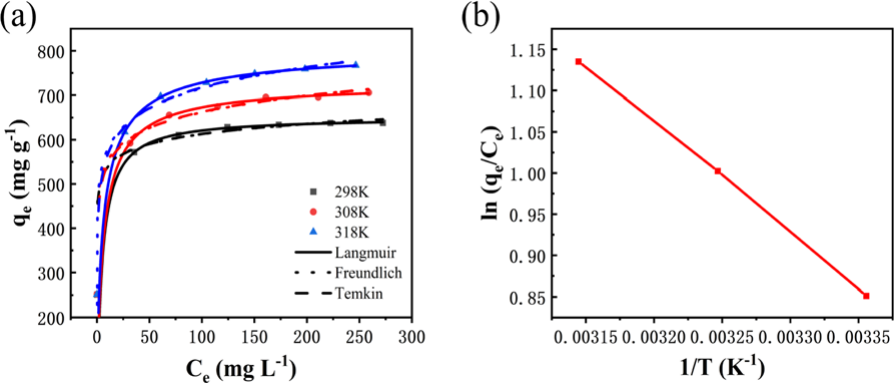
(a) Adsorption isotherm of MB on Fe_3_O_4_–N-BC, (b) thermodynamic fitting diagram.

Fig. 10(a) shows the effect of the initial dye concentration on the adsorption process of MB dye on Fe_3_O_4_–N-BC and the adsorption isotherm. At the test temperature, as the initial concentration increases, the adsorption amount of MB by Fe_3_O_4_–N-BC also increases until the adsorption equilibrium. The Langmuir (eqn (11)),^48^ Temkin (eqn (12)),^49^ and Freundlich (eqn (13))^50^ models were used to conduct isotherm fitting studies.11
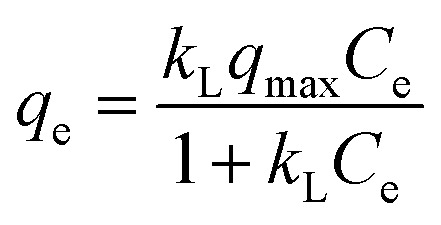
12
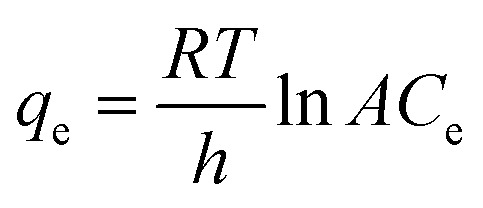
13
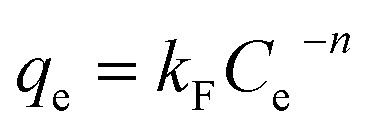
where *C*_e_ (mg L^−1^) is the dye liquor concentration at equilibrium, *q*_max_ (mg g^−1^) is the maximum adsorption capacity, *k*_L_ (L mg^−1^) is the Langmuir adsorption constant related to the adsorption capacity, *k*_F_ (L g^−1^) is the Freundlich adsorption constant related to the adsorption capacity, *n* is the Freundlich constant indicating the difficulty of the reaction, *R* is the gas constant 8.314 (J mol^−1^ K^−1^), *T* (K) is the temperature, *b* (J mol^−1^) is the Temkin constant, *A* (L g^−1^) is an equilibrium constant related to binding energy.

Analysis of the fitted isotherm parameters (referenced in Table S5†) reveals that both the Temkin and Freundlich models inadequately describe the adsorption behavior of MB by Fe_3_O_4_–N-BC, as indicated by their comparatively low correlation coefficients (*R*^2^). Conversely, the Langmuir model boasts the highest correlation coefficient, nearing unity. This finding suggests that the adsorption mechanism of MB onto Fe_3_O_4_–N-BC predominantly adheres to the Langmuir adsorption isotherm. Consequently, it can be inferred that the adsorption behavior of MB on the Fe_3_O_4_–N-BC surface entails uniform monolayer adsorption, rather than heterogeneous adsorption.^51^ The actual adsorption capacity of MB by Fe_3_O_4_–N-BC at 298 K is 638.08 mg g^−1^.

In comparison to biochar investigated in prior studies, the maximum adsorption capacity of Fe_3_O_4_–N-BC for MB, as displayed in Table 1, remains notably elevated. This observation underscores the effectiveness of Fe and N modification, signifying its promising potential for practical application.

**Table 1:** Comparison of maximum MB removal capacity of various biochar

Biochar materials	Kinetic model	Isotherm model	*q*_max_ (mg g^−1^)	References
Bamboo	Pseudo-second-order	Langmuir	210.86	45
Wakame	Pseudo-second-order	Langmuir	479.49	52
Chitosan flakes	Pseudo-second-order	Langmuir	143.53	53
Ashitaba biomass	—	Dubinin-Radushkevich	438.16	54
		Langmuir		
Banana peels	Pseudo-second-order	Langmuir	693.11	This work
Fe_3_O_4_–N-BC	Pseudo-second-order	Langmuir	651.47	This work
Aminated lignin	Pseudo-second-order	Freundlich	388.81	55

#### Adsorption thermodynamics analysis

In order to explore the effect of temperature on adsorption, thermodynamic analysis was performed. As can be seen from Fig. 10(b), as the adsorption temperature increases from 298 K to 318 K, the adsorption amount of MB gradually increases. In order to further study the relationship, the thermodynamic formula equations (eqn (14) and (15)). were used to perform thermodynamic fitting and research on the obtained data. The results can be seen from the figure. The corresponding thermodynamic parameter Gibbs freed the energy Δ*G*^0^, entropy change Δ*S*^0^, and enthalpy change Δ*H*^0^ are shown in the table.

14 Δ*G*^0^ = Δ*H*^0^ − *T*Δ*S*^0^

15
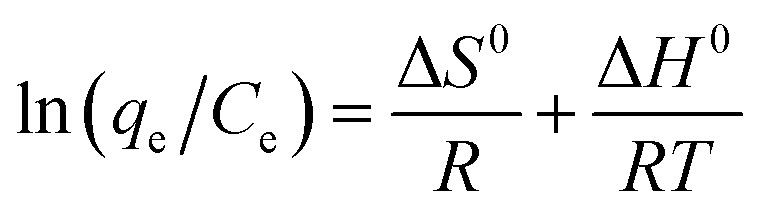
where Δ*G*^0^ (kJ mol^−1^) is the Gibbs free energy, Δ*S*^0^(J mol^−1^ K^−1^) is the entropy change, and Δ*H*^0^ (kJ mol^−1^) is the enthalpy change.

It can be seen from Table S6† that the positive enthalpy change (Δ*H*^0^, 11.184 kJ mol^−1^) indicates that MB adsorption on Fe_3_O_4_–N-BC is endothermic, and the positive entropy change (Δ*S*^0^, 44.623 J mol^−1^ K^−1^) indicates that the solid–liquid interface during the adsorption process disorder increases. The Gibbs free energy change of MB dropped from −2.1136 kJ mol^−1^ to −3.0061 kJ mol^−1^ within 298–318 K, which shows that this adsorption is a heat absorption process, and the adsorption of MB by Fe_3_O_4_–N-BC increases with the increase of temperature positive beneficial effects.

#### Recyclability

**Fig. 11:**
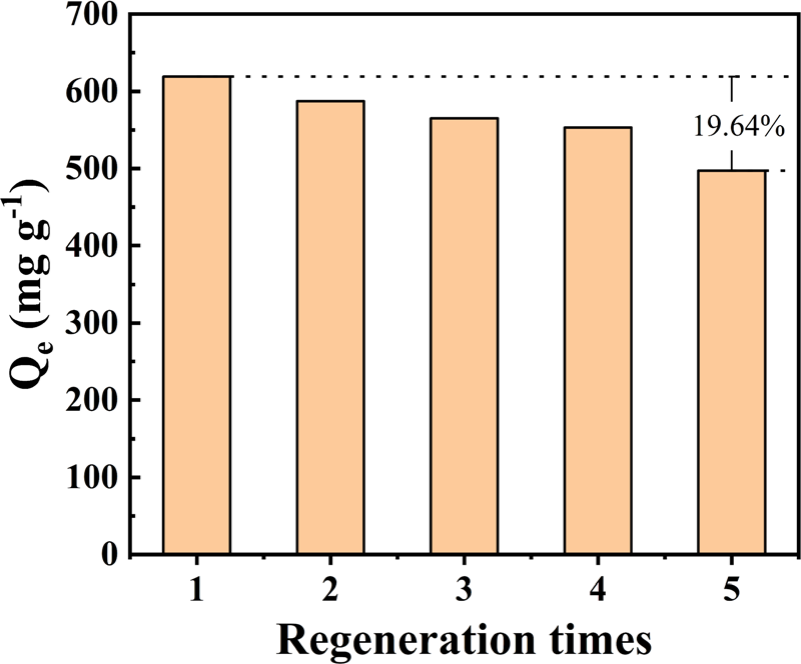
Regeneration performance.

Biochar loaded with Fe_3_O_4_ can be easily separated from water under external force, has a high recovery rate, and is easy to reuse, which is a crucial feature in adsorbent applications. The regeneration potential of Fe_3_O_4_–N-BC is elucidated in Fig. 11.

In the desorption trial, 0.1 mol L^−1^ HCl solution served as the eluent to extract the adsorbed MB from the biomass charcoal. After five adsorption–desorption times, the adsorption capacity of MB by Fe_3_O_4_–N-BC can still reach 497.18 mg g^−1^, accounting for 80.36% of the first adsorption capacity. The decrease in adsorption capacity with the number of cycles is due to the fact that MB on the surface of biomass charcoal permanently occupies some adsorption sites, resulting in some active sites being unavailable. Another reason is that part of the pore structure is blocked, resulting in fewer binding sites. The research results show that Fe_3_O_4_–N-BC has high stability and reusability, and has the potential to remove MB from wastewater.

#### Adsorption mechanism

**Fig. 12:**
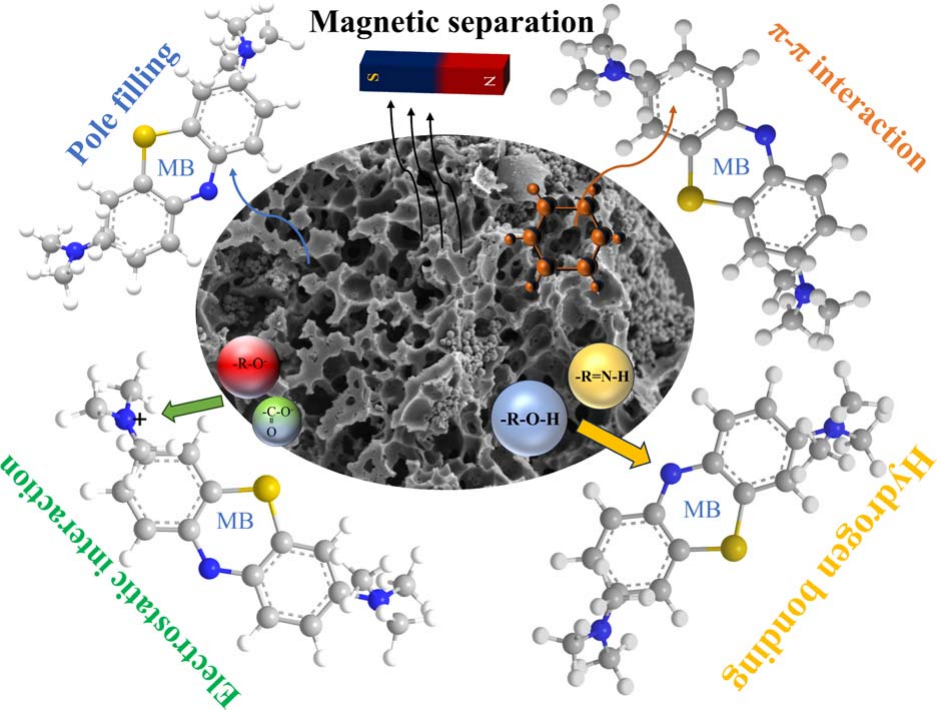
Schematic diagram of the adsorption mechanism of methylene blue on biochar.

Pore filling, electrostatic interaction, surface complex interaction, hydrogen bonding, pore diffusion, and π–π interaction are the mechanisms involved in MB adsorption by biomass carbon.^56,57^ Determining the adsorption mechanism is a challenging task because various factors often influence the adsorbent–adsorbate interaction simultaneously. The surface area, total pore volume, micropore volume, functional groups and hydrophilicity of biomass carbon will all affect its adsorption effect on MB. In order to study its adsorption mechanism, the adsorption of MB by biomass charcoal was analyzed and characterized. The specific surface area and pore volume of biomass charcoal hold pivotal significance in the adsorption of MB. Activation with potassium hydroxide notably augments both its specific surface area and total pore volume, consequently heightening its efficacy in adsorbing MB. Moreover, the biomass charcoal has a higher adsorption capacity after activation with potassium hydroxide. The carbon particles in material carbon can induce the formation of sp^3^ disordered or defective carbon and sp^2^ graphite during the activation process. The π–π bonds between the graphite lattice and methylene blue are improved due to the increased size and number of sp^3^ disorder. Due to the high defect density, Fe_3_O_4_–N-BC has specific active sites for MB adsorption. Despite the anticipated pore structure diminution resulting from iron-nitrogen co-modification, it offers the advantage of introducing additional functional groups and magnetic constituents. Various kinetic and equilibrium models were applied to the experimental dataset, revealing adherence to both the pseudo-second-order kinetic model and the Langmuir model. This suggests a significant contribution of chemical adsorption in the MB adsorption process by Fe_3_O_4_–N-BC.^58^ In essence, the principal method of removing MB using Fe_3_O_4_–N-BC involves pore filling and π–π interaction, succeeded by hydrogen bonding and electrostatic interaction. The adsorption mechanism is depicted in Fig. 12.

## Conclusions

In this study, N-modified banana peel biomass charcoal was generated by co-heating with urea at 700 °C and subsequently hydrothermal carbonization with Fe_3_O_4_ at 180 °C, with the calcination temperature, calcination time, and amount of activator KOH. The Fe_3_O_4_–N-BC substance was produced in 4 hours. The resulting materials were characterized using a variety of analytical techniques. The synthesized magnetic biomass charcoal material has a high specific surface area and magnetic characteristics, as well as good MB adsorption capability. At 298 K, Fe_3_O_4_–N-BC has the greatest adsorption capacity of 638.08 mg g^−1^ MB at adsorption equilibrium. The pseudo-second-order kinetic model and the Langmuir isotherm model are both capable of accurately describing the adsorption kinetics and isotherm. The experimental data suggest that the adsorption of MB onto Fe_3_O_4_–N-BC is characterized by homogeneous monolayer adsorption rather than heterogeneous adsorption. Furthermore, the adsorption process is dominated by chemisorption. Remarkably, the adsorbent maintained a high MB adsorption capacity of 497.18 mg g^−1^ even after five regeneration cycles. This work creates an MB adsorbent by recycling waste biomass resources, which not only contributes to the sustainable development of natural resources, but also broadens the potential application scope of biomass waste and aids in the resolution of dye wastewater and resource use issues. The strong adsorption performance of Fe_3_O_4_–N-BC on MB is predicted to be used for large-scale industrial wastewater treatment in the future.

## Data availability

The data supporting this article have been included in the main article and the ESI.†

## Author contributions

Conceptualization, Z.-X. G. and Y.-X. W.; methodology, Z.-X. G.; software, Z.-X. G., Y.-T. C., L.-Z. H.; writing – original draft preparation, Z.-X. G.; writing – review and editing, Z.-X. G., Y.-X. W., M. Steven., X.-J. Y., and X.-P. L.; project administration, X.-P. L.; funding acquisition, X.-P. L. All authors have read and agreed to the published version of the manuscript. All authors gave final approval for publication and agreed to be held accountable for the work performed therein. Collate acknowledgements in a separate section at the end of the article before the references and do not, therefore, individuals who provided help during the research (*e.g.*, providing language help, writing assistance or proof reading the article, *etc.*).

## Conflicts of interest

The authors declare no conflicts of interest.

## Supplementary Material

RA-014-D4RA04973J-s001

## References

[cit1] Katheresan V., Kansedo J., Lau S. Y. (2018). J. Environ. Chem. Eng..

[cit2] Kumar P. S., Varjani S. J., Suganya S. (2018). Bioresour. Technol..

[cit3] Monash P., Pugazhenthi G. (2009). Adsorption.

[cit4] Ma H., Li J.-B., Liu W.-W., Miao M., Cheng B.-J., Zhu S.-W. (2015). Bioresour. Technol..

[cit5] Wei Y., Li G., Wang C., Guo H. (2021). J. Colloid Interface Sci..

[cit6] Xia T., Ma Z., Ai M., Qian K., Zhu S., Rong M., Zhang P., Ye Y., Qin W. (2021). Chemosphere.

[cit7] Kashima K., Inage T., Yamaguchi Y., Imai M. (2021). J. Environ. Chem. Eng..

[cit8] Huo L.-Z., Guo C.-F., Gong Z.-X., Xu H., Yang X.-J., Wang Y.-X., Luo X.-P. (2024). Materials.

[cit9] Salazar R., Ureta-Zañartu M. S., González-Vargas C., do Nascimento Brito C., Martinez-Huitle C. A. (2018). Chemosphere.

[cit10] Mu B., Wang A. (2016). J. Environ. Chem. Eng..

[cit11] Illingworth J. M., Rand B., Williams P. T. (2019). Process Saf. Environ..

[cit12] Nasrullah A., Saad B., Bhat A., Khan A. S., Danish M., Isa M. H., Naeem A. (2019). J. Cleaner Prod..

[cit13] Sewu D. D., Boakye P., Woo S. H. (2017). Bioresour. Technol..

[cit14] Zhu Y., Gao J., Li Y., Sun F., Gao J., Wu S., Qin Y. (2012). J. Taiwan Inst. Chem. Eng..

[cit15] Li X., Wang C., Zhang J., Liu J., Liu B., Chen G. (2020). Sci. Total Environ..

[cit16] Thines K., Abdullah E., Mubarak N., Ruthiraan M. (2017). Renew. Sustain. Energy Rev..

[cit17] Du Q., Zhang S., Song J., Zhao Y., Yang F. (2020). J. Hazard. Mater..

[cit18] Duan X., Ao Z., Sun H., Zhou L., Wang G., Wang S. (2015). Chem. Commun..

[cit19] Wang X., Qin Y., Zhu L., Tang H. (2015). Environ. Sci. Technol..

[cit20] Wang G., Chen S., Quan X., Yu H., Zhang Y. (2017). Carbon.

[cit21] Leng L., Xu S., Liu R., Yu T., Zhuo X., Leng S., Xiong Q., Huang H. (2020). Bioresour. Technol..

[cit22] Liu S., Zhao C., Wang Z., Ding H., Deng H., Yang G., Li J., Zheng H. (2020). Chem. Eng. J..

[cit23] Li X., Jia Y., Zhou M., Su X., Sun J. (2020). J. Hazard. Mater..

[cit24] Zhou X., Liu Y., Zhou J., Guo J., Ren J., Zhou F. (2018). J. Taiwan Inst. Chem. Eng..

[cit25] Ai T., Jiang X., Liu Q., Lv L., Dai S. (2020). RSC Adv..

[cit26] Chen T., Luo L., Deng S., Shi G., Zhang S., Zhang Y., Deng O., Wang L., Zhang J., Wei L. (2018). Bioresour. Technol..

[cit27] Kim J.-K., Han Y.-K. (2008). Macromol. Res..

[cit28] Pourjavadi A., Ayyari M., Amini-Fazl M. (2008). Eur. Polym. J..

[cit29] Zhao H., Cheng Y., Lv H., Ji G., Du Y. (2019). Carbon.

[cit30] Mohanty P., Nanda S., Pant K. K., Naik S., Kozinski J. A., Dalai A. K. (2013). J. Anal. Appl. Pyrolysis.

[cit31] Zhang P., Tan X., Liu S., Liu Y., Zeng G., Ye S., Yin Z., Hu X., Liu N. (2019). Chem. Eng. J..

[cit32] Ranjithkumar V., Sangeetha S., Vairam S. (2014). J. Hazard. Mater..

[cit33] Liu P., Song Z., Miao L., Lv Y., Gan L., Liu M. (2024). Small.

[cit34] Qin Y., Jha S., Hu C., Song Z., Miao L., Chen Y., Liu P., Lv Y., Gan L., Liu M. (2024). J. Colloid Interface Sci..

[cit35] Mansuer M., Miao L., Qin Y., Song Z., Zhu D., Duan H., Lv Y., Li L., Liu M., Gan L. (2023). Chin. Chem. Lett..

[cit36] Duan X., O'Donnell K., Sun H., Wang Y., Wang S. (2015). Small.

[cit37] Rong X., Xie M., Kong L., Natarajan V., Ma L., Zhan J. (2019). Chem. Eng. J..

[cit38] Tang X., Ma S., Xu S., Yang Q., Huang Y., Wang J., Hua D. (2023). Chem. Eng. J..

[cit39] Sun Y., Wang T., Han C., Lv X., Bai L., Sun X., Zhang P. (2022). Bioresour. Technol..

[cit40] Liu B., Chen T., Wang B., Zhou S., Zhang Z., Li Y., Pan X., Wang N. (2022). J. Hazard. Mater..

[cit41] Zhou Y., Zhao X., Jiang Y., Ding C., Liu J., Zhu C. (2023). Sci. Total Environ..

[cit42] Volgmann K., Voigts F., Maus-Friedrichs W. (2010). Surf. Sci..

[cit43] Chen L., Huang Y., Zhou M., Xing K., Lv W., Wang W., Chen H., Yao Y. (2020). Chemosphere.

[cit44] Liu S., Li J., Xu S., Wang M., Zhang Y., Xue X. (2019). Bioresour. Technol..

[cit45] Sun X.-N., Yu K., He J.-H., Chen Y., Guo J.-Z., Li B. (2023). Bioresour. Technol..

[cit46] Chen Z.-L., Xu H., Bai L.-Q., Feng Y.-L., Li B. (2023). Prog. Nat. Sci.: Mater..

[cit47] Li B., Guo J., Lv K., Fan J. (2019). Environ. Pollut..

[cit48] Wang P., Cao M., Wang C., Ao Y., Hou J., Qian J. (2014). Appl. Surf. Sci..

[cit49] Saini K., Sahoo A., Biswas B., Kumar A., Bhaskar T. (2021). Bioresour. Technol..

[cit50] Zhang M., Gao B., Varnoosfaderani S., Hebard A., Yao Y., Inyang M. (2013). Bioresour. Technol..

[cit51] Brdar M., Šćiban M., Takači A., Došenović T. (2012). Chem. Eng. J..

[cit52] Yao X., Ji L., Guo J., Ge S., Lu W., Cai L., Wang Y., Song W., Zhang H. (2020). Bioresour. Technol..

[cit53] Marrakchi F., Ahmed M., Khanday W., Asif M., Hameed B. (2017). Int. J. Biol. Macromol..

[cit54] Xue H., Wang X., Xu Q., Dhaouadi F., Sellaoui L., Seliem M. K., Lamine A. B., Belmabrouk H., Bajahzar A., Bonilla-Petriciolet A. (2022). Chem. Eng. J..

[cit55] Wang C., Feng X., Shang S., Liu H., Song Z., Zhang H. (2023). Int. J. Biol. Macromol..

[cit56] Fan S., Wang Y., Li Y., Wang Z., Xie Z., Tang J. (2018). Environ. Sci. Pollut. Res..

[cit57] Peiris C., Gunatilake S. R., Mlsna T. E., Mohan D., Vithanage M. (2017). Bioresour. Technol..

[cit58] Yoon S.-Y., Lee C.-G., Park J.-A., Kim J.-H., Kim S.-B., Lee S.-H., Choi J.-W. (2014). Chem. Eng. J..

