# Supplementary material for: High adsorption to methylene blue based on Fe3O4–N-banana-peel biomass charcoal
Source: RSC Adv. 2024 Aug 15;14(35):25619–28. doi: 10.1039/d4ra04973j (PMC11325343; doi:10.1039/d4ra04973j)
Supplement: RA-014-D4RA04973J-s001 [file RA-014-D4RA04973J-s001.pdf]

Supplementary Information (SI) for RSC Advances.
This journal is © The Royal Society of Chemistry 2024

High adsorption to methylene blue based on Fe_3O_4-N-banana-peel biomass charcoal

Zhu-Xiang Gong[a], Mfitumucunguzi Steven[a], Yan-Ting Chen[a], Li-Zhu Huo[a], Hao Xu[a], Chao-Fei Guo[a], Xue-Juan Yang[a], Yu-Xuan Wang[a,*], Xi-Ping Luo[a,b,*]

a College of Chemistry and Materials Engineering, Zhejiang A&F University, Hangzhou 311300, China;
b Zhejiang Provincial Key Laboratory of Chemical Utilization of Forestry Biomass, Hangzhou 311300, China
* Corresponding authors.
E-mail addresses: 20190050@zafu.edu.cn (Y.-X. Wang), luoxiping@zafu.edu.cn (X.-P. Luo).

Table S1. Orthogonal design table

L_9 (4^3) orthogonal test design	Factors		
	(A) holding Temperature (min)	(B) carbonization temperature (℃)	(C) activator ratio (C/KOH)
1	A_1 60	B_1 600	C_1 1： 1
2	A_1 60	B_2 700	C_2 1： 2
3	A_1 60	B_3 500	C_3 1： 3
4	A_2 90	B_1 600	C_3 1： 3
5	A_2 90	B_2 700	C_1 1： 1
6	A_2 90	B_3 500	C_2 1： 2
7	A_3 120	B_1 600	C_2 1： 2
8	A_3 120	B_2 700	C_3 1： 3
9	A_3 120	B_3 500	C_1 1： 1

Figure S1. Methylene blue UV absorption spectrum

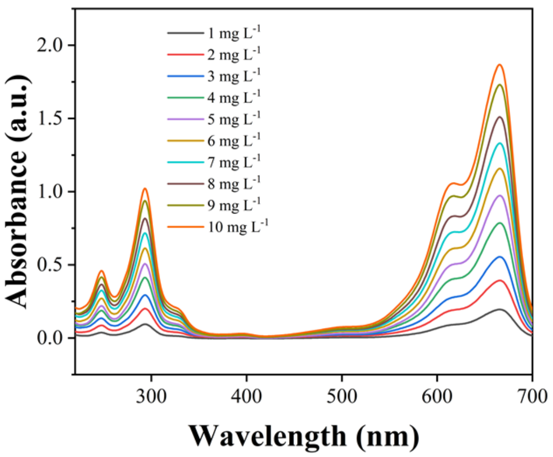

Figure S2. Methylene blue dye standard curve diagram

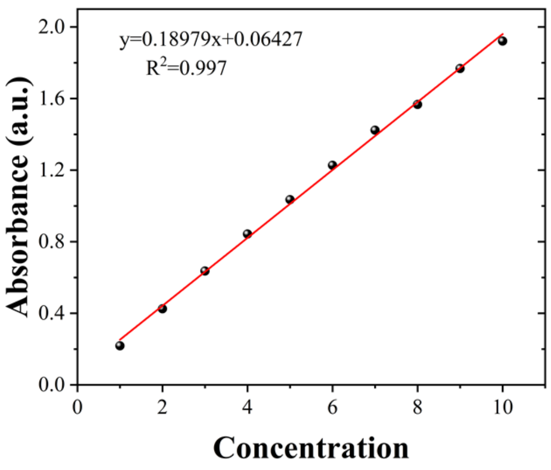

Table S2. Orthogonal experimental arrangements

L_9 (4^3) orthogonal test design	Factors		
	(A) holding Temperature (min)	(B) carbonization temperature (°C)	(C) activator ratio (C/KOH)
1	A_1 60	B_1 600	C_1 1：1
2	A_1 60	B_2 700	C_2 1：2
3	A_1 60	B_3 500	C_3 1：3
4	A_2 90	B_1 600	C_3 1：3
5	A_2 90	B_2 700	C_1 1：1
6	A_2 90	B_3 500	C_2 1：2
7	A_3 120	B_1 600	C_2 1：2
8	A_3 120	B_2 700	C_3 1：3
9	A_3 120	B_3 500	C_1 1：1
K_{i1}	185.56	173.29	158.71
K_{i2}	193.42	295.03	205.62
K_{i3}	184.04	94.70	198.69
k_{i1}	61.85	57.76	52.90
k_{i2}	64.47	98.34	68.54
k_{i3}	61.35	31.57	66.23
R_i	3.13	66.78	15.64
Primary and secondary sequence		B＞C＞A	
Excellent level	A_2	B_2	C_2
Optimal combination		$A_2B_2C_2$	

Table S3. Surface area and pore parameters of biochar.

Sample	BET surface area (m^2 g^{-1})	Pore volume (cm^3 g^{-1})	Average pore width (nm)
BC	1,827.9321	0.5198	2.7058
N-BC	1,616.3728	0.2416	3.1968
Fe_3O_4-N-BC	481.5964	0.097	3.6767

Table S4. Kinetics and intraparticle diffusion model parameters of MB adsorption on Fe_3O_4-N-BC

Model and parameters	pseudo-first-order dynamics		pseudo-second-order dynamics		intra-particle diffusion			
						I	II	III
Parameters	R^2	0.8316	R^2	0.9929	R^2	0.9778	5.7575	0.9779
	K_1	0.0397	K_2	0.000113	K_p	27.7533	5.7575	1.5598
	q_e	618.158	q_e	656.086	C	309.40	527.26	607.82

Table S5. Parameters of isotherm models for MB adsorption onto Fe_3O_4-N-BC.

T/K	Langmuir			Freundlich			Temkin		
	q_{max} (mg g^{-1})	K (L mg^{-1})	R^2	K	1/n	R^2	B (J mol^{-1})	A (L mg^{-1})	R^2
298	651.47	0.20169	0.9957	479.77	0.053	0.3649	32.84	1280.45	0.3652
308	721.14	0.14459	0.9912	460.69	0.078	0.5135	52.32	3.183	0.5142
318	785.26	0.13621	0.9942	466.50	0.092	0.6219	66.10	0.515	0.6230

Table S6. Thermodynamic fitting parameters.

Thermodynamic parameter	ΔH^0 (kJ mol^{-1})	ΔS^0 (J mol^{-1} K^{-1})	ΔG^0 (kJ mol^{-1})		
			298(K)	303(K)	313(K)
MB	11.184	44.623	-2.1136	-2.5598	-3.0061
